# Drug Exposure During Pregnancy: A Case-Control Study from a Primary Care Database

**DOI:** 10.1089/whr.2023.0123

**Published:** 2024-01-11

**Authors:** Ainhoa Gomez-Lumbreras, Marta Leston Vazquez, Carles Vilaplana-Carnerero, Oriol Prat-Vallverdu, Cristina Vedia, Rosa Morros, Maria Giner-Soriano

**Affiliations:** ^1^ Department of Pharmacotherapy, College of Pharmacy, University of Utah, Salt Lake City, Utah, USA.; ^2^ Àrea del Medicament i Servei de Farmàcia, Gerència d'Atenció Primària Barcelona Ciutat, Institut Català de la Salut, Barcelona, Spain.; ^3^ Universitat Autònoma de Barcelona, Bellaterra (Cerdanyola del Vallès), Spain.; ^4^ Fundació Institut Universitari per a la Recerca a l'Atenció Primària de Salut Jordi Gol i Gurina (IDIAPJGol), Barcelona, Spain.; ^5^ Plataforma SCReN, UIC IDIAPJGol, Barcelona, Spain.; ^6^ Marketing farmacéutico & Investigación clínica, Barcelona, Spain.; ^7^ Servei d'Atenció Primària Maresme, Barcelona, Spain.; ^8^ Institut Català de la Salut, Barcelona, Spain.

**Keywords:** abortion, induced, abortion, spontaneous, labor, obstetric, live birth, abnormalities, drug-induced, maternal-fetal exchange, case-control studies

## Abstract

**Objective:**

Drug exposure during pregnancy is frequent, even more during first trimester as pregnant women might not be aware of their condition. We used available electronic health records (EHRs) to describe the use of medications during the first trimester in pregnant women and to compare drug exposure between those women who had an abortion (either elective or spontaneous) compared to those who had live births.

**Materials and Methods:**

Case-control study of abortions, either elective or spontaneous (cases), and live birth pregnancies (controls) in Sistema d'Informació per al Desenvolupament de la Investigació en Atenció Primària (Catalan Primary Health electronic health records) from 2012 to 2020. Exposure to drugs during first trimester of pregnancy was considered to estimate the association with abortion by conditional logistic regression and adjusted by health conditions and other drugs exposure.

**Results:**

Sixty thousand three hundred fifty episodes of abortions were matched to 118,085 live birth pregnancy episodes. Cases had higher rates of alcohol intake (9.9% vs. 7.2%, *p* < 0.001), smoking (4.5% vs. 3.6%, *p* < 0.001), and previous abortions (9.9% vs. 7.8%, *p* < 0.001). Anxiety (30.3% and 25.1%, *p* < 0.001), respiratory diseases (10.6% and 9.2%, *p* < 0.001), and migraine (8.2% and 7.3%, *p* < 0.001), for cases and controls, respectively, were the most frequent baseline conditions. Cases had lower rate of drug exposure, 40,148 (66.5%) versus 80,449 (68.1%), *p* < 0.001. Association with abortion was found for systemic antihistamines (adjusted odds ratio [OR_adj_] 1.23, 95% confidence interval [CI] 1.19–1.27), antidepressants (OR_adj_ 1.11, 95% CI 1.06–1.17), anxiolytics (OR_adj_ 1.31, 95% CI 1.26–1.73), and nonsteroidal anti-inflammatory drugs (OR_adj_ 1. 63, 95% CI 1.59–1.67).

**Conclusions:**

These high rates of drug exposures during the first trimester of pregnancy highlights the relevance of informed prescription to women with childbearing potential.

## Introduction

The study of the use of drugs during pregnancy is mainly made by observational approaches, by analyzing pregnancy registries, case series, or cohort studies due to the ethics concerns of pregnant women's participation in clinical trials.^1^ During pregnancy, women can face chronic or acute illness, and the therapeutic approach is mainly based on clinical guidelines.^2^

Spontaneous abortion or miscarriage, described as the loss of pregnancy less than 20 weeks gestation, has not clearly been related to drug exposure as most of the times, a genetic factor lays as the main cause.^3^ Abortion can also be elective, decided by the pregnant woman if legally admitted, and can be induced, by medical decision. In Europe and North America, the unintended pregnancy rate is 35 per 1,000 women (aged between 15 and 49 years), the abortion rate of 17 per 1,000 women, but this rate increases up to 49% in the case of unintended pregnancies.^4^ Pregnant women could unintentionally be exposed to drugs and, according to the country legal basis, they may decide to have an elective abortion, which could be seen as a failure on the counseling when prescribing those drugs to women with childbearing potential.^5,6^

Special attention to drugs used in women with childbearing potential must be made, as exposure in the first trimester is often unintentional as they are unaware of their pregnancy. We aimed to assess the potential relationship of pregnant women's drug exposure and abortion during the first trimester of pregnancy.

## Materials and Methods

### Study design

This is a matched case-control study of pregnancy episodes to describe the first trimester of pregnancy drug exposure and assess the association with abortion.

### Study source

Sistema d'Informació per al Desenvolupament de la Investigació en Atenció Primària database characteristics have been described elsewhere.^7^ It contains electronic health records (EHRs) of the Primary Care Centers of the Catalan Health Institute (ICS), covering up to 6 million people and almost 500,000 pregnancy episodes. SIDIAP contains data also from the sexual and reproductive health care services (ASSIR) of the ICS. In Catalonia, most of the nonrisk pregnancies are followed at the ASSIR. Pregnancy episodes occurring during 2011–2020 were identified from the SIDIAP ASSIR module and through International Classification of Diseases 10th (ICD-10) diagnosis codes for gestation, abortion, and delivery (8–42 weeks) registered in the EHR.^8^

### Study population and matching

Pregnancy episodes in women from 12 to 50 years old were classified as cases (abortions) and as controls (pregnancies with a live-birth delivery), please see Case Definition section and Control Definition section. Data missing for pregnancy start date (PSD) and pregnancy end date (PED) were imputed based on average duration from the ASSIR data and final outcome for delivery or abortion was made based on clinical assumptions from data registered (*e.g.*, ICD 10th code registered for Encounter for supervision of normal pregnancy, unspecified, first trimester-Z34. 91, O03 spontaneous abortion, *etc*.).^8^

#### Case definition

Pregnancy episodes with a PSD, a PED, and an outcome for the end of pregnancy registered as “Abortion” within the first 120 days since the PSD. The outcome was not an ICD code, but an internal SIDIAP code for abortion, so no differences between elective or spontaneous abortion could be done.

#### Control definition

Pregnancy episodes with a date for the PSD and PED and outcome for the end of pregnancy as labor (“vaginal” or “C-section,” both included) were live-birth pregnancies (either full term or preterm birth).

Exclusion criteria were those pregnancies with no SIDIAP code for the PED even if the PSD was registered.

Cases were matched 1:2 to controls by mother's age by episode PSD (±3 years).

### Variables

Demographic characteristics, body mass index, smoking status, and alcohol intake were considered from 12 months before PSD up to 120 days after. Variables on medical conditions by ICD 10th diagnoses codes (to see those of interest please see Supplementary File S1) registered up to 120 after PSD. Previous episodes on live-birth pregnancies and abortions were considered if not occurring before the start of the study period (2011).

### Exposures

SIDIAP pharmacy invoice data were used to define drug exposure; any invoice occurring either 30 days before the PSD up to 120 days after this date or the date of abortion (whichever occurs first). Drugs were classified into the group according to the Anatomic Therapeutic Chemical classification level 3.^9^ Those drugs with less than a 3% case exposure or a different nonsignificant exposure (*p* > 0.05) were not included in the final analysis.

### Statistical analysis

Descriptive data, crude odds ratio (OR_crude_), and adjusted odds ratio (OR_adj_) with 95% confidence intervals (95% CI) were calculated using conditional logistic regression. Adjusted for these variables and for drug groups accounted for ≥3% of cases, for which the chi-square test showed significant differences (*p* < 0.05).

A sensitivity analysis for a subset of complete data- with no imputations - only of those pregnancies' records from the ASSIR with a spontaneous abortion label and deliver or C-section one for control was conducted. Clinical Trial Registration: EUPAS47450.

## Results

A total of 60,350 episodes of abortion were matched to 118,085 live-birth pregnancy episodes. Women's mean age was 33 years (interquartile range 28.9, 37.9). Higher rates of smoking and alcohol intake were registered for the cases (4.5% vs. 3.6%, *p* < 0.001 and 9.9% vs. 7.2%, respectively, *p* < 0.001). There were no differences on history of previous pregnancies with live-birth outcomes, although a higher rate of previous abortions was found for cases when compared to controls (9.9% vs. 7.8%, *p* < 0.001). Pregnant women characteristics at the pregnancy episodes are shown in Table 1. Cases had overall a higher prevalence of medical conditions, being anxiety the highest [18,268 (30.3%) compared to 29,614 (25.1%) in controls], followed by respiratory diseases [(6,410 (10.6%) and 10,915 (9.2%)] and migraine [(4,919 (8.2%) and 8,573 (7.3%)]. The overall drug exposure rate was higher among the controls [40,148 (66.5%) vs. 80,449 (68.1%), *p* < 0.001, cases].

**Table 1.** Descriptive Characteristics of the Pregnancy Episodes (Characteristics Are for the Pregnancy at That Specific Episode Accounting)

N* (%)*	*Abortions (*N* = 60,350)*	*Live-birth pregnancies (*N* = 118,085)*	
Complete data, no imputation	*N* = 48,733 (80.7)	*N* = 102,908 (87.1)	
Mother age at pregnancy episode (mean, IQR 25–75)	33.7 (28.9, 37.9)	33.5 (28.7, 37.5)	<0.001
Smoking (yes)	2,695 (4.5)	4,295 (3.6)	<0.001
Alcohol intake (yes)	5,993 (9.9)	8,552 (7.2)	<0.001
Obese (BMI = > 30 and/or Dx)	11,499 (19.1)	23,763 (20.1)	
MEDEA			<0.001
Rural	10,430 (17.3)	21,873 (18.5)	
Urban	6,134 (10.2)	11,101 (9.4)	
Level 1	6,630 (11.0)	12,173 (10.3)	
Level 2	8,029 (13.3)	15,821 (13.4)	
Level 3	8,523 (14.1)	16,314 (13.8)	
Level 4	9,519 (15.8)	18,388 (15.6)	
Level 5	11,040 (18.3)	22,360 (18.9)	
NA	45 (0.1)	55 (0.0)	
History of previous life births	4,721 (7.8)	9,476 (8.0)	0.138
History of abortions	5,970 (9.9)	9,220 (7.8)	<0.001
Diseases			
Mental disorders			
Anxiety	18,268 (30.3)	29,614 (25.1)	<0.001
Depression bipolar	4,590 (7.6)	7,060 (6.0)	<0.001
Eating disorders	2,926 (4.8)	4,553 (3.9)	<0.001
Personality disorder	446 (0.7)	556 (0.5)	<0.001
Psychosis	176 (0.3)	252 (0.2)	0.002
Cardiovascular			
Atrial fibrillation	17 (0.0)	22 (0.0)	0.263
Heart failure	10 (0.0)	17 (0.0)	0.881
Hypertension	958 (1.6)	1,478 (1.3)	<0.001
Ischemic heart disease	17 (0.0)	19 (0.0)	0.128
Myocarditis	1 (0.0)	6 (0.0)	0.488
Cerebrovascular disease	79 (0.1)	115 (0.1)	0.050
Diabetes mellitus	514 (0.9)	662 (0.6)	<0.001
Chronic kidney disease	55 (0.1)	54 (0.0)	<0.001
Neurologic diseases			
Epilepsy	390 (0.6)	599 (0.5)	<0.001
Migraine	4,919 (8.2)	8,573 (7.3)	<0.001
Immune mediated			
Immunodeficiencies	10 (0.0)	15 (0.0)	0.659
Lupus	26 (0.0)	43 (0.0)	0.582
Rheumatoid arthritis	163 (0.3)	293 (0.2)	0.412
Autoimmune thyroiditis	202 (0.3)	336 (0.3)	0.075
Transplant	16 (0.0)	25 (0.0)	0.590
Neoplasm	586 (1.0)	923 (0.8)	<0.001
Respiratory diseases	6,410 (10.6)	10,915 (9.2)	<0.001
HIV	37 (0.1)	34 (0.0)	0.002
Drug exposure			
No drug exposure	20,202 (33.5)	37,636 (31.9)	<0.001
Number of drugs			
1	14,740 (24.4)	30,570 (25.9)	
2–3	16,378 (27.1)	34,771 (29.4)	
4–5	6,175 (10.2)	11,138 (9.4)	
>5	2,855 (4.7)	3,970 (3.4)	

BMI, body mass index; HIV, human immunodeficiency virus; IQR, interquartile range; MEDEA, socioeconomic index.

The drug group with the highest rates of exposure was iodine therapy followed by those supplements indicated during pregnancy (vitamin B12 and folic acid and iron preparations). See Table 2 for specific active substance for each medication group, only most frequent active substance showed. The largest difference among drug exposure between cases and controls was for the Iodine therapy group (23.9% of use in cases and 35.2% in controls) and for nonsteroidal anti-inflammatory drugs (NSAIDs) (13.8% in cases and 6.1% in controls). Pregnancy episodes were most exposed to amoxicillin and fosfomycin among the rest of antibiotics of systemic use [10,119 (56.9%) and 11,344 (98.4%) cases and controls, respectively], and to omeprazole [4,028 (59.8%)] and ranitidine [2,160 (32.1%)] in the group of drugs for peptic ulcer and gastroesophageal reflux disease. Ibuprofen was the most used NSAID (9,598, 57.3%) and levonorgestrel and ethinylestradiol (2,170, 51.7%) among the systemic contraceptives group.

**Table 2.** Medication Groups and More Frequent Active Substances

Medication group	*Abortions exposure, *N* (%)*	*Live-birth pregnancies exposure, *N* (%)*	*Total exposure, *N* (%)*
Active substance			
Iodine therapy	14,407 (23.9)	41,525 (35.2)	
Potassium iodide + vitamin B12 + folic acid	13,424 (92.5)	37,733 (88.9)	51,157 (89.8)
Potassium iodide	1,080 (7.3)	4,702 (11.1)	5,782 (10.1)
Vitamin B12 and folic acid	7,877 (13.1)	21,422 (18.1)	
Folic acid	4,987 (62.0)	13,061 (59.4)	18,048 (60.1)
Cyanocobalamin, combinations	2,953 (36,7)	8,781 (39.9)	11,734 (39.1)
Iron preparations	4,781 (7.9)	12,502 (10.6)	
Ferrous sulfate	3,250 (65.7)	8,141 (62.1)	11,391 (63.1)
Ferrous glycine sulfate	860 (17.4)	2,844 (21.7)	3,704 (20.5)
Ferric proteinsuccinylate	530 (10.7)	1,295 (9.9)	1,825 (10.1)
Iron mannitol (ferrimanitol)	256 (5.2)	664 (5.1)	920 (5.1)
Other antibacterials	3,035 (5.0)	8,430 (7.1)	
Fosfomycin	3,004 (98.3)	8,340 (98.4)	11,344 (98.4)
Beta-lactam antibacterials, penicillins	5,420 (9.0)	11,344 (9.6)	
Amoxicillin	3,101 (53.9)	7,018 (58.3)	10,119 (56.9)
Amoxicillin and beta-lactamase inhibitor	2,402 (41.8)	4,524 (37.6)	6,926 (38.9)
Drugs for peptic ulcer and GORD	2,464 (4.1)	3,908 (3.3)	
Omeprazole	1,906 (73.5)	2,122 (51.2)	4,028 (59.8)
Ranitidine	363 (14.0)	1,797 (43.4)	2,160 (32.1)
Antihistamines for systemic use	2,905 (4.8)	3,567 (3.0)	
Cetirizine	948 (30.1)	1,105 (28.7)	2,053 (29.4)
Loratadine	680 (21.6)	896 (23.3)	1,576 (22.5)
Ebastine	691 (22.0)	762 (19.8)	1,453 (20.8)
Dexchlorpheniramine	296 (8.6)	526 (13.7)	795 (11.4)
Antidepressants	2,195 (3.6)	2,252 (1.9)	
Citalopram	462 (18.7)	454 (18.3)	916 (18.5)
Paroxetine	442 (17.9)	452 (18.2)	894 (18.01)
Fluoxetine	311 (12.6)	344 (13.9)	655 (13.2)
Sertraline	314 (12.7)	328 (13.2)	642 (13.0)
Escitalopram	220 (8.9)	213 (8.6)	433 (8.8)
Amitriptyline	217 (8.8)	200 (8.1)	417 (8.4)
Venlafaxine	135 (5.5)	139 (5.6)	274 (5.5)
Anxiolytics	3,147 (5.2)	2,506 (2.1)	
Diazepam	1,542 (44.5)	1,112 (41.5)	2,654 (43.2)
Alprazolam	802 (23.2)	645 (24.1)	1,447 (23.6)
Lorazepam	783 (22.6)	626 (23.4)	1,409 (23.0)
Anti-inflammatory and rheumatic products, nonsteroids (NSAIDs)	8,332 (13.8)	7,234 (6.1)	
Ibuprofen	4,974 (54.5)	4,624 (60.6)	9,598 (57.3)
Naproxen	1,449 (15.9)	1,316 (17.2)	2,765 (16.5)
Dexketoprofen	1,801 (19.7)	777 (10.2)	2,578 (15.4)
Diclofenac	691 (7.6)	678 (8.9)	1,369 (8.2)
Antiinfectives and antiseptics, excl combination with corticosteroids	2,717 (4.5)	7,383 (6.3)	
Clotrimazole	1,581 (52.6)	5,195 (63.7)	6,776 (60.7)
Fenticonazole	568 (18.9)	1,407 (17.2)	1,975 (17.7)
Clindamycin	334 (11.1)	614 (7.5)	948 (8.5)
Dequalinium	248 (8.2)	452 (5.5)	700 (6.3)
Hormonal contraceptives for systemic use	2,633 (4.4)	1,496 (1.3)	2,170 (51.7)
Levonorgestrel and ethinylestradiol	1,343 (49.9)	827 (54.9)	759 (18.1)
Desogestrel	401 (14.9)	358 (23.8)	486 (11.6)
Drospirenone and ethinylestradiol	362 (13.4)	124 (8.2)	396 (9.4)
Dienogest and ethinylestradiol	284 (10.5)	112 (7.4)	231 (5.5)
Etonogestrel	208 (7.7)	23 (1.5)	

Only medication groups with over 3% (either cases or controls) frequency of exposure during the first trimester of the pregnancy episodes and statistical difference (*p* < 0.05).

GORD, gastroesophageal reflux disease; NSAIDs, nonsteroidal anti-inflammatory drugs.

The OR_adj_ for any drug exposure was 1.014 (1.010–1.019), *p* < 0.001 when compared to nonexposure. For the drug groups that showed a statistically significant different exposure among cases and controls and a frequency of exposure higher than 3% the OR_crud_ and OR_adj_ are shown in Table 3. Association was found for antihistamines for systemic use [OR_adj_ (95% CI) 1.23 (1.19–1.27)], antidepressants [1.11 (1.06–1.17)], anxiolytics [1.31 (1.26–1.73)], NSAIDs [1.63 (1.59–1.67)] and hormonal contraceptives for systemic use [1.71 (1.65–1.78)]. The results of the sensitivity analysis did not differ from the ones using all data. Results for the sensitivity analysis conducted only with those pregnancy episodes defined by the complete data nonimputed can be seen in Supplementary File S2.

**Table 3.** Odds Ratios for Association with Abortion for Each of the Medication Groups

Medication group (ATC code)	OR_crude_ (95% CI)	OR_adj_ (95% CI)
Iodine therapy (H03C)	0.69 (0.68–0.70)	0.71 (0.69–0.72)
Folic acid and derivatives (B03B)	0.76 (0.75–0.78)	0.77 (0.75–0.79)
Iron preparations (B03A)	0.80 (0.78–0.82)	0.86 (0.84–0.89)
Other antibacterials (J01X)	0.77 (0.74–0.80)	0 81 (0.78–0.84)
Beta-lactam antibacterials, penicillins (J01C)	0.95 (0.93–0.98)	0.87 (0.84–0.89)
Drugs for peptic ulcer and GORD (A02B)	1.14 (1.09–1.19)	0.98 (0.94–1.02)
Antihistamines for systemic use (R06A)	1.34 (1.29–1.39)	1.23 (1.19–1.27)
Antidepressants (N06A)	1.46 (1.40–1.52)	1.11 (1.06–1.17)
Anxiolytics (N05B)	1.67 (1.61–1.73)	1.31 (1.26–1.73)
Anti-inflammatory and rheumatic products, nonsteroids (NSAIDs) (M01A)	1.67 (1.63–1.71)	1.63 (1.59–1.67)
Anti-infectives and antiseptics, excl combination with corticosteroids (G01A)	0.79 (0.76–0.82)	0.83 (0.79–0.86)
Hormonal contraceptives for systemic use (G03A)	1.95 (1.87–2.02)	1.71 (1.65–1.78)

Adjusted by: Anxiety, bipolar depression, eating disorder, migraine, respiratory diseases, MEDEA index, obesity (BMI > = 30 and diagnosis), alcohol intake, smoking, or previous abortions, and completed pregnancies.

ATC, anatomic therapeutic chemical classification of drugs; CI, confidence interval; OR_adj_, adjusted odds ratio; OR_crude_, crude odds ratio.

## Discussion

This case-control study shows a high rate of exposure to drugs during first trimester of pregnancy comparing those pregnancies ending in abortion to those ending in a live birth. Hormonal contraceptives for systemic use, NSAIDs, anxiolytics, systemic antihistamines, and antidepressants showed association with abortion. Among the group of drugs studied, the supplements such as iodine or folic acid showed no relationship with abortion.

The use of these supplements (iodine therapy, folic acid and derivatives, and iron preparations) is supported by most of the pregnancy guidelines.^10^ Planned pregnancies are more likely to adhere to pregnancy recommendations and, women with chronic medical conditions are more likely to adhere to these, supporting the protective association of these supplements.^11^

Among the anti-infectives, our results agree with previous studies, beta-lactams are the most used during pregnancy, but no risk of spontaneous abortion has been shown for beta-lactams, while for quinolones, tetracyclines, sulfonamides, metronidazole, and macrolides, this potential risk has been defined.^12,13^

No association was found for drugs used to treat peptic ulcer and gastroesophageal reflux, histamine 2 receptor antagonist (H_2_RA), or proton pump inhibitors (PPI). Considering heartburn is one of the most common complaints during pregnancy and that up to now no other studies have found any risk of abortion for PPI or H_2_RA, we think these results can support the safe use of these during the pregnancy.^14^

Our study shows association to abortion for the first trimester exposure to antihistamines for systemic use. There are controversial results on the association between early exposure to antihistamines and malformations.^15,16^ Contrary to our results, meta-analysis of Etwel et al. did not find any association between the first trimester exposure to H_1_ antihistamines and abortion.^15^ In a German study on chronic diseases in women with childbearing potential, the prevalence of allergies (either systemic or dermatological) was up to 11.3%, and exposure to antihistamines in pregnant women has been described up to 1%, as they are also indicated in some other medical conditions such as urticaria, dermatitis, pruritus, rhinitis, nausea, and motion sickness, making any concern about their safety during pregnancy a priority.^17^

Central nervous system drugs use has increased among women with childbearing potential, and no clear recommendations on their continuing use during pregnancy exist, as also negative outcomes have been described among those women with untreated depression during pregnancy.^18^ Our results in a Catalan population are in line with the ones published by the Base de datos para la Investigación Farmacoepidemiológica en Atención Primaria database that showed a risk for abortion for the use of antidepressants and anxiolytics during pregnancy, and the metanalysis by Xing et al. found similar risk of abortion for antidepressants.^19,20^ Two observational studies in Danish population did not find any risk for mirtazapine or duloxetine specifically, neither one for fluoxetine with American population.^21,22^ Women can already be on antidepressants or anxiolytics when becoming pregnant or initiate these treatments during the pregnancy, and up to now no clear conclusions to guide their use during pregnancy have been established.

We must acknowledge that elective abortion due to negative pregnancy outcomes described while on central nervous system drugs, even more if an unplanned pregnancy, can be biasing the association we found, as it is also acknowledged by the authors of the Danish population observational studies.

During pregnancy, up to 10% of women experience primary headaches such as migraine and tension headache as a cause of the hormonal fluctuation, and also women with rheumatic diseases during pregnancy may need analgesia.^23^ NSAIDs, mainly represented by ibuprofen, naproxen, and diclofenac showed association between the first trimester exposure and abortion. The latest meta-analysis on the early exposure in pregnancy to NSAIDs and abortion confirms our results.^24^ However, the rates of NSAIDs exposure, as these are often in use in an “as needed” basis or as over the counter (OTC) drugs make it difficult to be accurate assessing their exposure.^25^

Up to 40% of women with potential childbearing have reported not to be using contraception and abortion rates have increased in the last decades.^4,26^ For women with chronic conditions, induced abortion rates have demonstrated to be similar to those without diseases, so if we focus only on drug exposure, our results raise the concern of unplanned pregnancies and elective abortion.^27^ Because high-income countries have lower rates of unplanned pregnancies but higher rates of abortion, our findings on the association between abortion and contraceptives may be explained by women not wanting to become pregnant and contraceptive failure or fear to undesired birth outcomes once exposed.

Our results show that drug prescription in women with childbearing potential is very important and may lead to better family planning information. Thus, it may be necessary to advert women with childbearing potential on the risks of use of drugs.

As it may be necessary to advert women on the potential risks of use of drugs, more studies are needed, not only about the specific knowledge of potential teratogenic effects of some drugs but also on the effects of suspending or changing a chronic treatment during pregnancy on the women's and on the infants' health, or about the safety of drugs during pregnancy. This information obtained in research would directly impact on the clinical practice.

This study has two important strengths to highlight. First, it has been conducted in a database containing information on pregnancy duration and ending causes and also relied in an algorithm using obstetrics ICD-10 codes diagnoses. These codes have been used previously to identify pregnancy episodes and their duration.^28–30^ Our results did not change for the sensitivity analysis, using only those pregnancies with complete ASSIR data. Second, we defined the population at a pregnancy episode level, although we did not make any differences between new users and prevalent ones, but we did consider previous pregnancies outcomes (abortion or stillbirth).

This study has some limitations. Those regarding the accuracy of data register in EHRs have been already defined and some specific to the topic.^31^ Abortion in EHRs is not consistently recorded and also different models for its register protecting women's privacy may be difficult, the correct classification of abortion in spontaneous, elective, or induced, and the outcome registered in SIDIAP did not specify the abortion type, so cases could be spontaneous abortions or induced/elective ones. As an example, in the ASSIR, the emergency contraception is recorded in a module to what SIDIAP has not access to and, these are not prescribed and dispensed in community pharmacies, so there is no information on emergency contraception. We might have underregister supplements or NSAIDs as these are OTC drugs in Catalonia.

Exposure definition was at an invoice level, we did not have information on the strength, so the amount of exposure could not be quantified. We cannot rule out that a potential indication bias, as women in chronic therapies such as antidepressants, or not willing pregnancy, in contraceptives, may elect the abortion, this we cannot specify if women with unplanned pregnancy may choose intentional abortion were on these medications. However, in the case of antihistamines for systemic use, this association should be studied deeply as this group of drugs is not pregnancy or chronic disease related.

## Conclusions

Use of drugs during the first trimester of pregnancy, when women may not be aware that they are pregnant, is common making necessary to inform women in childbearing age of the risk of use of drugs during pregnancy. Association with abortion for frequently used drugs such as NSAIDs, antihistamines, and central nervous system need to be further investigated. The potential confounding bias in our study highlights the importance of a good adherence to contraceptives and an improvement in contraception plans.

## Supplementary Material

Supplemental data

Supplemental data

## Registration

EUPAS37675 (https://www.encepp.eu/encepp/viewResource.htm?id=37676). AEMPS classification EPA-OD (IDI-BRO-2020-21, September 16, 2020).

## Abbreviations Used

95% CI: 95% confidence interval
ASSIR: sexual and reproductive health care services
ATC: anatomic therapeutic chemical classification of drugs
BMI: body mass index
EHR: electronic health records
GORD: gastroesophageal reflux disease
H_2_RA: histamine 2 receptor antagonist
ICD-10: International Classification of Diseases 10th version
ICS: Catalan Health Institute
MEDEA: socioeconomic index
NSAIDs: nonsteroidal anti-inflammatory drugs
OR_adj_: adjusted odds ratio
OR_crude_: crude odds ratio
OTC: over the counter
PED: pregnancy end date
PPI: proton pump inhibitors
PSD: pregnancy start date
SIDIAP: Sistema d'Informació per al Desenvolupament de la Investigació en Atenció Primària

## References

[1] Sarayani A, Albogami Y, Thai TN, et al. Prenatal exposure to teratogenic medications in the era of risk evaluation and mitigation strategies. Am J Obstet Gynecol 2022;227(2):263.e1–263.e38; doi: 10.1016/j.ajog.2022.01.004 35032444

[2] Centers for Disease Control and Prevention. Treating for Two: Medicine and Pregnancy: Guidelines and Recommendations for Treating and Managing Health Conditions During Pregnancy. CDC; 2022. Available from: https://www.cdc.gov/pregnancy/meds/treatingfortwo/treatment-guidelines.html [Last accessed: July 27, 2022].

[3] Hassold TJ. A cytogenetic study of repeated spontaneous abortions. Am J Hum Genet 1980;32(5):723–730. 7424911 PMC1686106

[4] Bearak J, Popinchalk A, Ganatra B, et al. Unintended pregnancy and abortion by income, region, and the legal status of abortion: Estimates from a comprehensive model for 1990–2019. Lancet Glob Health 2020;8(9):e1152–e1161; doi: 10.1016/S2214-109X(20)30315-6 32710833

[5] Elsinga J, de Jong-Potjer LC, van der Pal-de Bruin KM, et al. The effect of preconception counselling on lifestyle and other behaviour before and during pregnancy. Womens Health Issues 2008;18(6):S117–S125; doi: 10.1016/j.whi.2008.09.003 19059545

[6] Zomerdijk IM, Ruiter R, Houweling LMA, et al. Isotretinoin exposure during pregnancy: A population-based study in The Netherlands. BMJ Open 2014;4(11):e005602; doi: 10.1136/bmjopen-2014-005602 PMC4244495 25392022

[7] Recalde M, Rodríguez C, Burn E, et al. Data Resource Profile: The Information System for Research in Primary Care (SIDIAP). Int J Epidemiol 2022;51(6):e324–e336; doi: 10.1093/ije/dyac068 35415748 PMC9749711

[8] Lestón Vázquez M, Vilaplana-Carnerero C, Gomez-Lumbreras A, et al. Drug exposure during pregnancy in primary care: An algorithm and observational study from SIDIAP database, Catalunya, Spain. BMJ Open 2023;13(8):e071335; doi: 10.1136/bmjopen-2022-071335 PMC10445402 37607789

[9] WHO Collaborating Centre for Drug Statistics Methodology. ATC/DDD Index 2022. Norwegian Institute of Public Health; 2022. Available from: https://www.whocc.no/atc_ddd_index/ [Last accessed: September 2, 2023].

[10] World Health Organization. Nutritional Interventions Update: Multiple Micronutrient Supplements During Pregnancy. Published July 29, 2020. Available from: https://www.who.int/publications/i/item/9789240007789 32783435

[11] De Wolff MG, Johansen M, Rom AL, et al. Degree of pregnancy planning and recommended pregnancy planning behavior among women with and without chronic medical conditions—A large hospital-based cross-sectional study. Acta Obstet Gynecol Scand 2021;100(6):1051–1060; doi: 10.1111/aogs.14069 33368141

[12] Muanda FT, Sheehy O, Bérard A. Use of antibiotics during pregnancy and risk of spontaneous abortion. CMAJ 2017;189(17):E625–E633; doi: 10.1503/cmaj.161020 28461374 PMC5415390

[13] Omranipoor A, Kashanian M, Dehghani M, et al. Association of antibiotics therapy during pregnancy with spontaneous miscarriage: A systematic review and meta-analysis. Arch Gynecol Obstet 2020;302(1):5–22; doi: 10.1007/s00404-020-05569-4 32409925

[14] Li CM, Zhernakova A, Engstrand L, et al. Systematic review with meta-analysis: The risks of proton pump inhibitors during pregnancy. Aliment Pharmacol Ther 2020;51(4):410–420; doi: 10.1111/apt.15610 31909512

[15] Etwel F, Faught LH, Rieder MJ, et al. The risk of adverse pregnancy outcome after first trimester exposure to H1 antihistamines: A systematic review and meta-analysis. Drug Saf 2017;40(2):121–132; doi: 10.1007/s40264-016-0479-9 27878468

[16] Kar S, Krishnan A, Preetha K, et al. A review of antihistamines used during pregnancy. J Pharmacol Pharmacother 2012;3(2):105–108; doi: 10.4103/0976-500X.95503 22629082 PMC3356948

[17] Kersten I, Lange AE, Haas JP, et al. Chronic diseases in pregnant women: Prevalence and birth outcomes based on the SNiP-study. BMC Pregnancy Childbirth 2014;14(1):75; doi: 10.1186/1471-2393-14-75 24552439 PMC3943445

[18] Cohen LS. Relapse of major depression during pregnancy in women who maintain or discontinue antidepressant treatment. JAMA 2006;295(5):499; doi: 10.1001/jama.295.5.499 16449615

[19] Kitchin Á, Huerta C, Llorente-García A, et al. The role of prenatal exposure to antidepressants, anxiolytic, and hypnotics and its underlying illness on the risk of miscarriage using BIFAP database. Pharmacoepidemiol Drug 2022;31(8):901–912; doi: 10.1002/pds.5488 PMC9543237 35689300

[20] Xing D, Wu R, Chen L, et al. Maternal use of antidepressants during pregnancy and risks for adverse perinatal outcomes: A meta-analysis. J Psychosom Res 2020;137:110231; doi: 10.1016/j.jpsychores.2020.110231 32889478

[21] Ankarfeldt MZ, Petersen J, Andersen JT, et al. Duloxetine exposure during pregnancy and the risk of spontaneous and elective abortion: A Danish Nationwide Observational Study. Drugs Real World Outcomes 2021;8(3):289–299; doi: 10.1007/s40801-021-00252-9 34008161 PMC8324661

[22] Ostenfeld A, Petersen TS, Pedersen LH, et al. Mirtazapine exposure in pregnancy and fetal safety: A nationwide cohort study. Acta Psychiatr Scand 2022;145(6):557–567; doi: 10.1111/acps.13431 35320582 PMC9321713

[23] On Behalf of the European Headache Federation School of Advanced Studies (EHF-SAS), Negro A, Delaruelle Z, et al. Headache and pregnancy: A systematic review. J Headache Pain 2017;18(1):106; doi: 10.1186/s10194-017-0816-0 29052046 PMC5648730

[24] Ying X-H, Bao D-N, Jiang H-Y, et al. Maternal non-steroidal anti-inflammatory drug exposure during pregnancy and risk of miscarriage: A systematic review and meta-analysis. Eur J Clin Pharmacol 2022;78(2):171–180; doi: 10.1007/s00228-021-03222-w 34635936

[25] Zafeiri A, Mitchell RT, Hay DC, et al. Over-the-counter analgesics during pregnancy: A comprehensive review of global prevalence and offspring safety. Hum Reprod Update 2021;27(1):67–95; doi: 10.1093/humupd/dmaa042 33118024

[26] Cea Soriano L, Asiimwe A, Van Hemelrijck M, et al. Feasibility study to identify women of childbearing age at risk of pregnancy not using any contraception in The Health Improvement Network (THIN) database. BMC Med Inform Decis Mak 2020;20(1):164; doi: 10.1186/s12911-020-01184-0 32682423 PMC7368731

[27] Venne K, Scott S, Bernatsky S, et al. Induced abortions in women with systemic lupus erythematosus. Lupus 2021;30(3):484–488; doi: 10.1177/0961203320979741 33323010

[28] Andrade SE, Shinde M, Moore Simas TA, et al. Validation of an ICD -10-based algorithm to identify stillbirth in the Sentinel System. Pharmacoepidemiol Drug Saf 2021;30(9):1175–1183; doi: 10.1002/pds.5300 34089206

[29] Margulis AV, Palmsten K, Andrade SE, et al. Beginning and duration of pregnancy in automated health care databases: Review of estimation methods and validation results: Pregnancy beginning and duration in automated data. Pharmacoepidemiol Drug Saf 2015;24(4):335–342; doi: 10.1002/pds.3743 25627986

[30] Minassian C, Williams R, Meeraus WH, et al. Methods to generate and validate a Pregnancy Register in the UK Clinical Practice Research Datalink primary care database. Pharmacoepidemiol Drug Saf 2019;28(7):923–933; doi: 10.1002/pds.4811 31197928 PMC6618019

[31] Lupattelli A, Wood ME, Nordeng H. Analyzing missing data in perinatal pharmacoepidemiology research: Methodological considerations to limit the risk of bias. Clin Ther 2019;41(12):2477–2487; doi: 10.1016/j.clinthera.2019.11.003 31791674

