# Supplementary material for: Drug Exposure During Pregnancy: A Case-Control Study from a Primary Care Database
Source: Womens Health Rep (New Rochelle). 2024 Jan 11;5(1):13–21. doi: 10.1089/whr.2023.0123 (PMC10798141; doi:10.1089/whr.2023.0123)
Supplement: Supplemental data [file Suppl_FileS1.docx]

**ICD 10^th^ Diagnoses codes for medical conditions of interest**

Anxiety F40*-F48*

Atrial fibrillation I48*

Mental disorders F00*-F09*

Psychosis F20*-F29*

Depression/Bipolar F30*-F39*

Personality disorder F60*-F69*

Eating disorder F50*-F59*

Ischemic heart disease I20*-I25*

Rheumatoid arthritis M05*-M09*

Lupus L93*

Diabetes mellitus E10*-E14*

Immunodeficiencies D80*

Autoimmune thyroiditis E06.3

Epilepsy G40*

Hypertension I10*-I15*

Heart failure I50*

Cerebrovascular disease I60*-I69*, G45*-G46*

Chronic kidney disease N18*

Respiratory diseases J40*-J47*, J60*-J99*, G47.3

Migraine G43*

Myocarditis I40*-I41*

Neoplasia C00*-C97*

Obesity E66*

Transplant Z94*

HIV B20*-B24*
