# Supplementary material for: Drug Exposure During Pregnancy: A Case-Control Study from a Primary Care Database
Source: Womens Health Rep (New Rochelle). 2024 Jan 11;5(1):13–21. doi: 10.1089/whr.2023.0123 (PMC10798141; doi:10.1089/whr.2023.0123)
Supplement: Supplemental data [file Suppl_FileS2.docx]

Table 2s. Sensitivity analysis for complete pregnancies data from ASSIR. Odds ratio for the therapeutic groups

| **Medication group (ATC code)** | **OR (95%CI)** | **OR adj (95%CI)** | **p** |
| --- | --- | --- | --- |
| Iodine therapy (H03C) | 0.9 (0.87-0.92) | 0.9 (0.88-0.93) | <0.001 |
| Folic acid and derivatives (B03B) |  |  |  |
| [Iron preparations](https://www.whocc.no/atc_ddd_index/?code=B03A&showdescription=no) (B03A) | 0.76 (0.73-0.8) | 0.76 (0.72-0.8) | <0.001 |
| [Other antibacterials](https://www.whocc.no/atc_ddd_index/?code=J01X&showdescription=no) (J01X) | 0.78 (0.74-0.83) | 0.79 (0.74-0.83) | <0.001 |
| Beta-lactam Antibacterials, Penicillins (J01C) |  |  |  |
| Drugs for Peptic Ulcer and Gastro-Esophageal Reflux Disease (GORD) (A02B) |  |  |  |
| [Antihistamines for systemic use](https://www.whocc.no/atc_ddd_index/?code=R06A&showdescription=no) (R06A) | 1.3 (1.22-1.38) | 1.19 (1.11-1.26) | <0.001 |
| Antidepressants (N06A) |  |  |  |
| Anxiolytics (N05B) | 1.52 (1.43-1.62) | 1.32 (1.23-1.41) | <0.001 |
| Antiinflammatory and antirheumatic products, non-steroids (M01A) | 1.7 (1.64-1.77) | 1.65 (1.59-1.72) | <0.001 |
| Antiinfectives and Antiseptics, Excl Combination with Corticosteroids (G01A) |  |  |  |
| Hormonal contraceptives for systemic use (G03A) | 1.82 (1.69-1.96) | 1.68 (1.56-1.8) | <0.001 |

Grey: these therapeutic groups did not show a frequency of exposure of 3% or more/statistically significance
